# Modified Procedure of Noncultured Epidermal Suspension Transplantation: Changes are the Core of Vitiligo Surgery

**DOI:** 10.4103/0974-2077.79192

**Published:** 2011

**Authors:** Anantha Prasad Holla, Ravinder Kumar, Davinder Parsad, AJ Kanwar

**Affiliations:** *Vitiligo Institute for Care and Research, Mangalore, Karnataka, India*; 1 *Post Graduate Institute of Medical Education and Research, Chandigarh, India*

**Keywords:** Vitiligo surgery, non cultured epidermal suspension transplantation, modified procedure

## Abstract

Epidermal cell suspensions are being increasingly used in the surgical management of vitiligo. The procedure suffers from several drawbacks such as high cost of the procedure, difficulty of procuring the reagents etc. We hereby describe modifications which allow the use of cheaper, more commonly available alternatives.

## INTRODUCTION

Vitiligo is a common acquired disorder of pigmentation characterized by sharply defined white patches of variable shape and dimensions. The condition may be psychologically devastating due to the stigma attached to it. Since ‘beauty is skin deep’, a white patch of vitiligo is of great cosmetic concern especially in darker skin.

The pathogenesis of vitiligo remains unclear, but several findings strongly suggest that melanocyte destruction may be caused by different pathogenetic mechanisms.[1] Although medical therapy has improved considerably over the years, at present, complete repigmentation cannot be achieved in all the treated patients.[2] Since 1952, several surgical methods have been developed for stable vitiligo.[3] Some of these procedures which aim to replenish lost melanocytes have gained popularity in recent time. These methods offer replacement of destroyed melanocytes but do not cure the underlying cause of vitiligo.

The technique of noncultured epidermal suspension was pioneered by Gauthier *et al*.[4] They stated that this technique could emerge as a simple and effective alternative to the costly cultured melanocyte transplantation technique. Olsson and Juhlin who pioneered the Swedish procedure of melanocyte transplantation first used the melanocyte medium for the suspension of the noncultured melanocytes.[5]

However, several practical problems are associated with noncultured melanocyte transplantation. Significant run-off of the suspension from the recipient site was associated with the high fluidity of the suspension and because of this reason cellular suspension transplantation is not successful in difficult-to-treat and uneven areas. High cost and concern of mitogenesis are drawbacks of the usage of the melanocyte medium.[6] Moreover, standard procedures of noncultured epidermal transplantation described in the literature are not foolproof and there is a scope for improvement and modification. We are proposing a new modified procedure with following modifications as an effort to overcome the drawbacks of earlier procedures.

## MODIFICATIONS

### 1. Using phosphate buffered saline instead of the melanocyte medium for the preparation of the melanocyte suspension

Phosphate buffered saline (PBS) is a buffer solution commonly used in biological research. The osmolarity and ion concentrations of the solution usually match those of the human body (isotonic) and it is nontoxic to cells. We propose that, since PBS helps in the prevention of osmosis-induced cell death, it will help in melanocyte survival and negate the need for melanocyte media. With this, the cost of the procedure can be reduced since there is no need of melanocyte media and there are no concerns of mitogenesis due to the melanocyte medium.

### 2. Avoiding the use of trypsin inhibitor in the process of preparation of noncultured epidermal suspension

Trypsinized skin graft is washed with PBS instead of treating with a trypsin inhibitor solution. We found that there was neither significant effect on the outcome nor any increase in adverse events by doing so. Moreover, this reduces the cost of the procedure.

### 3. Use of chlorhexidine gauze

Initially few drops of the suspension are placed on the denuded area; over this sterile chlorhexidine gauze cut in the shape of the denuded area is placed. More suspension is spread over this; due to surface tension of the suspension placed earlier, an even layer of suspension will be formed over the denuded area. The run-off of the suspension and the need for the solutions to increase the viscosity of suspension are avoided. This modification will help in putting the melanocyte suspension over uneven surfaces. Since it also helps in holding cells over the denuded area, good results can be expected and it is useful in treating difficult and large areas. We have used chlorhexidine gauze in several melanocyte procedures and found out that it does not have a detrimental effect on melanocyte survival. Plain sterile paraffin gauze can be an alternative. But chlorhexidine has an added advantage of antibacterial action, so we preferred it. Because of the greasy nature of the gauze, it did not affect the wound healing as we never observed the problem of adhesion of gauze to wound and difficulty in wound healing in any of our patients.

### 4. Using meshed collagen sheet or a sterile gauze smeared with placental extract gel

It is said that a moist wound heals faster compared to a dry wound.[7] We used meshed collagen sheet as biological dressing to achieve a ‘moist wound healing’; additionally it ensures the adherence of melanocytes to the denuded area as observed in various previous studies. Further, mesh also ensures that underlying melanocytes are with constant contact with PBS, which is there in the overlying gauze. Since there is a concern about the risk of prion disease with the use of collagen which is of bovine origin, we tried an alternate option where a sterile gauze smeared with the placental extract gel was used instead of a collagen sheet.[8] We found out that both achieved a comparable outcome. We used the placental extract gel to achieve a ‘moist wound healing’; we believe that any plain gel or even a gauze soaked in PBS could also be other alternative.

It is well established that noncultured epidermal suspension transplantation is highly surgeon dependent. Methods used by a particular surgeon may not give similar efficacy when used by another. Moreover, no procedure is perfect and changes are the essence of science. There is a need for more and more research and modifications in this particular procedure to achieve results which help to alleviate the agony of patients with psychologically devastating disease. We believe that the modifications described here will be useful to make this procedure easy, safe, economical, and effective in the management of stable vitiligo, particularly in difficult-to-treat, uneven and larger areas.
